# Foreign Language Enjoyment and Anxiety as the Correlates of the Ideal L2 Self in the English as a Foreign Language Context

**DOI:** 10.3389/fpsyg.2021.790648

**Published:** 2021-11-25

**Authors:** Jalil Fathi, Farnoosh Mohammaddokht

**Affiliations:** ^1^Department of Applied Linguistics, University of Kurdistan, Sanandaj, Iran; ^2^TEFL, University of Kurdistan, Sanandaj, Iran

**Keywords:** FLE, ideal L2 self, EFL, motivation, FLCA

## Abstract

Learners’ emotions in learning a foreign language are claimed to shape complicated dynamic associations contributing to their motivational and linguistic outcomes, as evidenced by recent research in this area. In order to advance this research area, this study sought to investigate the foreign language enjoyment (FLE) and foreign language classroom anxiety (FLCA) as the predictors of ideal L2 self in Iranian English as a Foreign Language (EFL) context. The total number of 195 English-major students from various universities completed an online survey containing the three scales in question. The measurement models were first verified using confirmatory factor analysis (CFA). Then, the structural model for the relations among the variables was tested employing structural equation modeling (SEM). The SEM results showed that although FLE and FLCA significantly predicted ideal L2 self, FLE was a stronger predictor of ideal L2 self than FLCA. This study provides significant pedagogical implications for EFL practitioners.

## Introduction

Research into the field of teaching and learning has shown an outstanding shift from teacher-centered toward student-centered education (Blumberg and Pontiggia, 2011). In light of this paradigm shift, there has been a dramatic change of attention from teachers toward students. As Hismanoglu (2000) maintained, more emphasis has been put on learning and learners rather than on teaching and teachers. As English as a Foreign Language (EFL) is progressing toward learner-centered pedagogy (Emaliana, 2017), investigating how EFL students learn and what variables impact their learning has become the center of attention of numerous researchers in different contexts (Khamkhien, 2010; Nomnian and Arphattananon, 2018; Derakhshan et al., 2021; Esra and Sevilen, 2021; Pishghadam et al., 2021). With growing enthusiasm in the role of learners in language learning, it is crucial to understand what factors drive different experiences for different students. In association with factors influencing learning, various learner variables, such as attitudes (Petrides, 2006), anxiety, and motivation (Khodadady and Khajavy, 2013), foreign language enjoyment (FLE; Jin and Zhang, 2018), and ideal L2 self (Dörnyei, 2010) are believed to play a significant role in foreign language (FL) achievement.

The ideal L2 self, mainly conceptualized as “the L2-specific facet of one’s ideal self” (Dörnyei, 2009, p. 29), has been identified as a variable increasing motivated L2 learning behavior (Dörnyei, 2009). A key component of a L2 motivational self system of Dörnyei (2009), the ideal L2 self refers to the ideal image held in learners’ mind of the type of L2 user they hope to become in the future (Magid and Chan, 2012; Nourzadeh et al., 2020; Yousefifard and Fathi, 2021). For instance, if individuals desire to be competent L2 speakers, the imaginary picture of their L2 self acts as a strong motive force for learning the L2 since they want to minimize the difference between their ideal and actual selves (Dörnyei, 2010). Moreover, research has revealed that ideal L2 self is correlated with different variables such as willingness to communicate (Khajavy and Ghonsooly, 2017), and enthusiastic employment of the foreign language (Papi et al., 2019). According to Dörnyei (2009), individuals desire to reach a state in which their self-concept (beliefs about oneself) corresponds with their self-guides’ (e.g., ideal self). Previous studies have reported that learners who visualize a clearer ideal L2 self would demonstrate a greater degree of L2 learning motivation (e.g., Kim and Kim, 2014).

Along with the rise of humanistic psychology, students’ *affect* (emotion) as well as its influence on language learning has received much attention (Dörnyei, 2010). It was first Arnold (1999) who called for L2 researchers’ attention toward the important concept of *affect* inside the classroom. With great emphasis on learners’ affection, a plethora of studies have looked into the role of emotions in L2 learning (e.g., Gregersen et al., 2014; Dewaele, 2019). In addition to negative emotions, according to Wang et al. (2021), positive emotions, including grit, academic engagement, enjoyment, emotion regulation, well-being, and other similar constructs can influence different aspects of life, especially L2 teaching and learning. With the emergence of positive psychology (Seligman and Csikszentmihalyi, 2000), the field which explores how individuals thrive and flourish, researchers have recognized the significance of positive emotions in the domain of L2 learning (Jiang and Li, 2017; Wang et al., 2021). As MacIntyre and Gregersen (2012) stated positive emotions can contribute to learners’ L2 development.

Studies with more holistic view on negative and positive classroom emotions have shown that anxiety impedes students’ cognitive processing, while positive emotions contribute to broadening their cognitive capacity (see Dewaele and MacIntyre, 2014). Foreign language classroom anxiety (FLCA) and FLE are recognized as two influential variables in predicting L2 outcomes (e.g., Dewaele and Dewaele, 2017) and FL learning (Dewaele and MacIntyre, 2014). During past decades, a number of L2/FL scholars have shown their interest in exploring the effect of FLCA and FLE on general English courses (Elahi-Shirvan and Taherian, 2018), learners’ performance (Dewaele and Alfawzan, 2018), gender (Dewaele et al., 2016), and their WTC (Dewaele, 2019). Nevertheless, a trace of ideal L2 self is not evidenced in their studies. One example of such investigations is the work of Dewaele (2019), in which students’ WTC was negatively and strongly predicted by FLCA, while positively by FLE. This suggests that learner’s WTC benefits from increased FLE and low levels of FLCA in the classroom. Similarly, Dewaele and Dewaele (2018) argued that FLCA is a significant negative correlate of students’ WTC, whereas learners’ positive perceptions toward the FL are its positive predictors. Research in this area has revealed that FLA is a negative predictor of learning and communication (Gregersen and MacIntyre, 2014), whereas enjoyment broadens learners’ perspective about learning language (MacIntyre and Gregersen, 2012) and influences their hardiness and resilience positively (Dewaele et al., 2019). Enjoyment has been also found to be correlated with L2 achievement (Papi and Khajavy, 2021).

Reviewing the existing literature of L2 learning has also shown that FLE and FLCA are interrelated (Dewaele and MacIntyre, 2016). Despite the array of published literature on the impact of FLCA and FLE on L2 learning, few empirical studies have explored such constructs in the field of EFL with the particular concentration on ideal L2 self (Elahi-Shirvan et al., 2018; Papi and Khajavy, 2021). As a result, it seems to be a dearth of studies investigating the role of FLCA and FLE in predicting ideal L2 self among EFL students. Put differently, it is still not verified whether and in what way FLCA and FLE may influence ideal L2 self. Therefore, in order to fill the raised gaps and to shed more light on the role of FLE and FLCA in predicting ideal L2 self, the aim of this study was set to examine the joint effect of FLE and FLCA on ideal L2 self among Iranian EFL students. By having the combination of these variables together, this study adds novelty to the current literature.

## Literature Review

### Foreign Language Classroom Anxiety and Foreign Language Enjoyment

Broadly speaking, anxiety refers to a kind of aversive emotional and motivational state, which arouses in intimidating circumstances (Eysenck et al., 2007). In the field of L2 learning, an increasing research attention has been directed to anxiety as a negative emotion (Horwitz, 2001; Lee, 2018; Teimouri et al., 2019). As MacIntyre and Mercer (2014) noted, anxiety is the most extensively studied emotion among negative emotions in L2 research. To date, many studies have examined the association of anxiety with learners’ performance and achievement (e.g., Horwitz, 2010; Dewaele, 2017). Moreover, the study of Horwitz (2010), showed an inverse correlation between FL anxiety and proficiency scores. As far as L2 learning is concerned, the seminal work of Horwitz et al. (1986) added a new era in research regarding anxiety by developing FLCA scale. Furthermore, they claimed that foreign language anxiety (FLA) and general anxiety are strongly correlated. With the advent of emotion research in L2, FLA has been the target of attention due to its significant influence on students’ cognitive L2 performance (Zhou et al., 2020) and consequences for L2 outcomes (Jin and Zhang, 2018).

Explorations in this line of research have revealed that FLA influences academic achievement negatively (e.g., Khodadady and Khajavy, 2013; Botes et al., 2020). Chen and Chang (2004), for instance, argued that anxious individuals suffer from language difficulty, show poor development skills, and achieve low grades. In conjunction with the role of FLA in learning, one example is a study of Hu et al. (2021), which focused on exploring the correlation between primary school students’ FLA and their FL achievement. For data collection, FLCA scale was administered to measure FLA and their FL achievement was assessed through low-stakes assessments plus high-stakes formal examinations. Findings revealed that participants’ FL achievement was predicted by FLA inversely. The results also confirmed a stronger correlation for high-stakes formal examinations in comparison to low-stakes regular assessments.

Concerning the variables that play a role in affecting FLA, Alamer and Almulhim (2021) have underscored the role of motivation in determining learners’ anxiety level. The results demonstrated a negative correlation between general language anxiety and autonomous motivation in learning English. In addition, Aslan and Thompson (2021) investigated the relationship between Turkish EFL learners’ beliefs and language anxiety. Findings revealed that Turkish EFL students’ negative attitudes toward English and their classroom performance anxiety were positively associated with fear of ambiguity.

Delving deeply in this respect, Jiang and Dewaele (2020) examined the role of sociobiographical variables and language variables in predicting Chinese university students’ FLA in English. Findings indicated that FLA was significantly correlated with frequent use of language, experience abroad, geographical background, self-perceived oral competence, age of onset of acquisition, and language achievement level. Further results of this study suggested that FLA can be observed as much outside the classroom as inside, while different sources are involved. Regarding the occurrence of remote and online language learning in recent years, Russell (2020) argued that during the global COVID-19 pandemic both teachers and language learners were likely to experience a considerable amount of general anxiety. Given that online language students are required to have interaction with their teacher and classmates through audio and video tools in the target language, they may struggle with anxiety that stems from both the language and the use of the instructional technologies while communicating (Pichette, 2009). Reviewing the literature regarding anxiety, Russell (2020) noted some techniques and interventions that have been suggested to instructors in order to reduce students’ level of anxiety; Teachers can (a) offer the students advice on effective use of language learning strategies, especially during online learning, (b) ask learners to express their fears either in groups or through journaling, (c) encourage students by posting motivational messages and ensure them they have their teachers’ supports and guidance, (d) devote some virtual office time to guide and tutor new online learners, and (e) make students realize that making mistakes is a sign of learning not a failure.

While a bulk of studies were carried out to investigate negative emotions such as FLA, inadequate research attention had been paid to positive emotions before the arrival of positive psychology in L2 learning (MacIntyre and Gregersen, 2012). The mounting interest in positive psychology in L2 studies (Dewaele et al., 2019) has led to the probing into positive emotions such as enjoyment in FL learning process (Li et al., 2018). Therefore, a call has been made to deepen our understanding of what role positive emotions could play in facilitating the development of language learning (MacIntyre and Gregersen, 2012).

The recent years have witnessed a turn of research into positive emotions, including growth mindset (Zarrinabadi et al., 2021), joy (Tahmouresi and Papi, 2021), well-being (Zeng et al., 2016), and grit (Teimouri et al., 2020). To this list, enjoyment is the widely studied positive emotion (Li, 2020; Li et al., 2021). Particularly, FLE, which could be conceptualized as enjoyment experienced during L2 learning, has drawn increasing attention since it is recognized as the counterpart of FLA (Jiang and Dewaele, 2019). FLE scale, which was initially introduced by Dewaele and MacIntyre (2014), has remained the most approved for measuring FLE. In order to balance research literature that has examined the role of negative emotions, language anxiety in particular, researchers have taken into consideration the role of positive emotions (Sadoughi and Hejazi, 2021). According to Cao (2014), both negative and positive emotions are dominant individual variables that influence classroom interactions. In addition, Dewaele and MacIntyre (2016) have emphasized that researchers should investigate the role of positive emotions, specifically enjoyment in learning.

In line with research into negative and positive emotional effects, an accumulated research interest into the combined effect of FLCA and FLE on learning has been evident during the past years (e.g., Li et al., 2020, 2021). A study of Dewaele and MacIntyre (2014) was the first study which investigated FLCA and FLE at the same time and underscored the importance of both positive and negative emotions. This mixed-method study collected data from 1,746 FL learners with different ages from all over the world. By and large, participants showed higher degrees of FLE than FLCA. Their research findings confirmed that a range of learner-external and learner-internal variables predicted learners’ level of FLE and FLCA in the FL classroom. Findings indicated that FLE is higher in those older learners who have higher degree of education, feel more proficient that their classmates, and know several languages. Another finding of this study is that female participants showed higher FLCA and FLE, compared to male participants. It is also proven that as learners mastered FL their level of FLA decreased, while their FLE increased. Moreover, the majority of participants mentioned that speaking the target of language in front of their peers was both enjoyable and highly anxiety-provoking. In another study, Dewaele and Alfawzan (2018) examined FLE and FLA among 189 L2 students in London and EFL learners in Saudi Arabia. Findings revealed that learners’ L2 performance was positively influenced by FLE and negatively by FLA.

Previous research has shown that FLCA is negatively associated with WTC (Liu and Jackson, 2008), frequent use of language learning strategy, perceived competence (MacIntyre et al., 1997), and FL achievement (Hu et al., 2021). In turn, FLE is positively linked with WTC (Dewaele and Pavelescu, 2021), more positive perceptions toward L2 use, and the amount of time learners spend speaking the FL (Dewaele et al., 2018), and language proficiency (Jin and Zhang, 2018). Furthermore, a quite recent study conducted by Zhang et al. (2020) highlighted the significance of positive psychology, particularly FLE in learning FL/L2 learning. The results of their study demonstrated that FLE has a mediating role in the association between motivation and L2/FL proficiency.

Recently, Dewaele and Dewaele (2020) investigated the effect of teachers on FLCA and FLE at a single point at time. In other words, they intended to find out whether students’ FLE and FLCA were similar when learning the same FL in the classes of two different teachers. Participants were divided to two groups: a group of students had two FL teachers, while the rest of them were taught by just one FL teacher. Findings revealed that whereas students reported significant higher levels of FLE with the main teacher, FLCA stayed constant with both teachers. The positive and significant FLE with the main teacher was predicted by the teacher’s frequent use of the target language, unpredictability, and students’ perceptions toward their teacher.

Even though FLA and FLE represent negative and positive emotions in the FL classroom, it is worth mentioning that they are not opposite of each other (MacIntyre and Gregersen, 2012). Rather, according to Dewaele and MacIntyre (2016), FLA and FLE can be regarded as the right and left feet of the language learners. Furthermore, the findings from a subsequent study by Dewaele and MacIntyre (2019) confirmed that FLCA and FLE are fairly two independent emotions and relatively separate dimensions. The results of their study showed an average inverse relationship between FLE and FLCA. In a same vein, Dewaele and MacIntyre (2014) asserted that anxiety and enjoyment are independent emotions that the presence of one does not imply the absence of the other.

### Ideal L2 Self

The primary research into L2 motivation was largely influenced and inspired by the studies of Robert Gardner (the Canadian social psychologists) and his colleagues (e.g., Gardner and Lambert, 1972; Gardner, 1985). Retrospectively, a socio-educational model of Gardner (1985), which was a classic conceptualization of the integrative motive, had prevailed in most concepts of the L2 motivation. Starting in the 1990s, the construct of *integrativeness* was bombarded with criticisms despite its significance (Dörnyei, 2010). For instance, in FL learning contexts where students learn a FL just as a curriculum subject without much exposure to the target language (e.g., learning English as a FL in China) integration does not make sense. Given that the model of Gardner (1985) was hardly applicable to educational contexts, especially EFL context, (Dörnyei, 2005, 2009) proposed a new theory of the L2 motivational self-system built upon the theoretical foundation of Gardner (1985). This construct, including ideal L2 self, ought-to L2 self, and L2 experience, has provided a wider scope for other variables to be examined within L2 motivation and can be applied extensively across various linguistic and cultural contexts (Dörnyei, 2010). During the past decades, inquiries have arisen to investigate the two core components of proposed construct of Dörnyei (2005, 2009), namely ought-to L2 self and the ideal L2 self in different L2 settings (see Taguchi et al., 2009). The ideal L2 self, which has been considered as an important notion to uncover and realize motivation regarding language learning (e.g., Kim and Kim, 2012), is referred to as a favorable self-image, which L2 learners want to reach in the future with regard to learning the L2 (Dörnyei, 2005, 2009). The ideal L2 self, according to Dörnyei (2010) acts as a powerful motivator when one tries to bridge the gap and distance between their actual L2 skills and the future ambitions of L2 learning. In recent years, numerous researchers have carried out research to illuminate the link between deal L2 self and FLE (e.g., Ryan, 2009; Yashima, 2009; Ueki and Takeuchi, 2013). The significance of ideal L2 self in affecting motivational intensity and persistence in L2 learning is manifested in the study of Feng and Papi (2020).

In regard to the effect of ideal L2-self on language achievement, Dörnyei and Chan (2013) claimed that highly motivated learners with more distinct ideal L2 self-images have the potential of achieving L2 more successfully. Moreover, Kong et al. (2018) probed into the association between ideal L2 self and FLE in Korean EFL context. Findings of their study indicated that learners who had greater ideal L2 self seemed to enjoy their L2 learning. It was also revealed that although ideal L2-self played an important role in affecting FLE, ought-to L2 self had no correlation with learners’ view of L2 learning.

Concerning L2 motivational self system in association with L2 anxiety, findings from the study of Papi (2010) confirmed the significant role of ideal L2 self in influencing L2 anxiety. It was also noted that L2 motivational self system encourages students to devote more effort and energy to learning English. Similarly, Ueki and Takeuchi (2013) pointed to the negative contribution of ideal L2 self to anxiety. Their proposed model indicated that ideal L2 self had a significant effect on L2 learning motivation. In another study, Peng (2015) highlighted that ideal L2 self was inversely related to L2 anxiety. In contrast, the subsequent investigation of Yang (2012), done in Taiwan, showed a different picture regarding the inter-connection between ideal L2 self and anxiety. This study recruited 108 undergraduate students in Applied English who had experience of learning EFL for at least 6years. The findings revealed that ideal L2 self affected anxiety significantly. Concerning the association between enjoyment and ideal L2 self, Papi and Khajavy (2021) highlighted that ideal L2 self was a positive correlate of enjoyment. In a same vein, Teimouri (2017) emphasized the association between joy and ideal L2 self.

Reviewing the abovementioned studies has indicated that the majority of research has either compared and contrasted FLE and FLA or explored their association with different learner variables. Yet, no empirical study has so far dealt with the role of both FLE and FLCA in predicting ideal L2 self. Nor has any study examined the constructs of ideal L2 self, FLE, and FLCA. The only published study, which has partially touched this area, is a study of Tahmouresi and Papi (2021). The purpose of their study was to find out how learners’ L2 writing selves (e.g., ought L2 self and ideal L2 self) directly and indirectly influence L2 writing achievement through anxiety and enjoyment. Taken together, the findings of the presents study will contribute to the exiting literature related to anxiety, enjoyment, and motivation.

## Materials and Methods

### Participants

A total number of 195 English-major learners from several universities in Iran responded to the survey. The respondents were undergraduate English major students from different provinces and they were selected based on convenience sampling procedure (Ary et al., 2018). The sample consisted of both male (*N*=88) and female (*N*=107) students whose ages ranged from 21 to 29, with mean age of 22.16. Their English learning experience varied from 5 to 14years, with an average of 6.3years of experience.

### Instruments

#### Foreign Language Enjoyment Scale

This scale contained 10 items which were taken from Jiang and Dewaele (2019). These statements indicate both the social and private components of FLE (Dewaele and MacIntyre, 2016). Every item was measured using a five-point Likert scale varying from “not at all” to “very much so.”

#### Foreign Language Classroom Anxiety Scale

This scale which was developed by Jiang and Dewaele (2019) consisted of eight items concerning physical symptoms of anxiety, nervousness, and lack of confidence. Jiang and Dewaele (2019) adapted the items from the FLCAS (Horwitz et al., 1986). The scale measures two components of low anxiety (two items) and high anxiety (six items). Reverse-coding was applied to items of low anxiety for the sake of consistency in computing the total score.

#### Ideal L2 Self Scale

The Ideal L2 Self scale included eight items which were adapted from Papi and Abdollahzadeh (2012). This scale intended to assess the learners’ image of their ideal selves regarding using English. Each item was measured using a Likert scale ranging from 1 (Strongly Disagree) to 6 (Strongly Agree).

### Procedure

As the design of the study was a correlational research, the data were gathered by giving the three self-report measuring instruments of the three constructs (i.e., FLE, FLCA, and ideal L2 self). In so doing, the questionnaires were put together in a form of an online survey using the Google Docs application.^1^ The data collection started in the winter of 2021 with cooperation of some English-major university instructors who were teaching at different universities in Iran. The link of the Google-Docs survey was sent to these instructors. Then they shared the links with their English major undergraduate students *via* Telegram or WhatsApp groups and requested them to respond to the items of the questionnaires. It took about a month to collect all the data.

### Data Analysis

The data were analyzed using SPSS (version 22) and AMOS (21) for data imputation, descriptive statistics analyses, and carrying out confirmatory factor analysis (CFA) as well as Structural Equation Modelling (SEM). The latent constructs were validated using CFA (Kline, 2011). Then SEM was employed as a powerful multivariate procedure to verify the hypothesized structural model. As the first step, normality of the data, outliers, and missing values were checked by an initial screening. Expectation–maximization algorithm was used for missing data (Kline, 2011). Univariate outliers were examined with standard scores, and Mahalanobis D2 was employed to determine multivariate outliers. Following recommendations by Tabachnick and Fidell (2007), univariate and multivariate outliers were detected and eliminated, leading to 195 valid cases for SEM analyses. Skewness and kurtosis values fell within the range of −1 to +1, confirming the normality of the data.

The validity of the measurement models for the three latent constructs was examined using CFA and fit indices (Kline, 2011). In the current study, *χ*^2^/*df* (chi-square to degrees of freedom ratio), goodness-of-fit index (GFI), Tucker-Lewis index (TLI), comparative fit index (CFI), and root mean square error of approximation (RMSEA). The acceptable values of the indices are *χ*^2^/*df*<3, GFI>0.95, TLI>0.95, CFI>0.95, and RMSEA<0.06 (Hu and Bentler, 1999). As the measurement models for the FLE and did not demonstrate adequacy to the data, two items were removed from the FLE scale. Then AMOS 21 was employed to investigate the structural model with the maximum likelihood technique and variance–covariance matrices as input.

## Results

Before running SEM for testing the structural model, the results of CFAs indicated the validity of the measurement models (see Table 1). As indicated in Table 1, the models showed acceptable fit. In other words, the construct validity of the three scales (i.e., FLCA, FLE, and ideal L2 self) was approved. Also, Cronbach’s α calculations indicated that the scales were of acceptable reliability indices. Table 2 indicates the reliability of the scales.

**Table 1:** Fit indices of the measurement models.

	*χ* ^2^	*df*	*χ*^2^/*df*	GFI	CFI	TLI	RMSEA
FLE	40.23	20	2.01	0.96	0.98	0.97	0.05
FLCA	212.11	107	1.98	0.97	0.98	0.98	0.04
Ideal L2 self	98.12	59	1.66	0.96	0.97	0.97	0.05

**Table 2:** Reliability of the scales.

Scales	Cronbach’s α
FLE	0.81
FLCA	0.79
Ideal L2 self	0.85

After that, descriptive statistics and correlations between the constructs were calculated. Table 3 indicates the descriptive statistics and correlations among FLCA, FLE, and ideal L2 self. As illustrated in Table 3, the correlation between FLE and ideal L2 self (*r*=0.56, *p*<0.01) was greater than the correlation between FLCA and ideal L2 self (*r*=0.44, *p*<0.01).

**Table 3:** Descriptive statistics and correlations.

	Mean (SD)	Correlation			
		1	2	3	4
FLCA	2.36 (0.79)	1.00			
FLE	3.28 (1.02)	−0.34^*^	1.00		
Ideal L2 self	3.19 (0.98)	−0.44^**^	0.56^**^	1.00	

* *p*<0.05;

** *p*<0.01.

**Figure 1:**
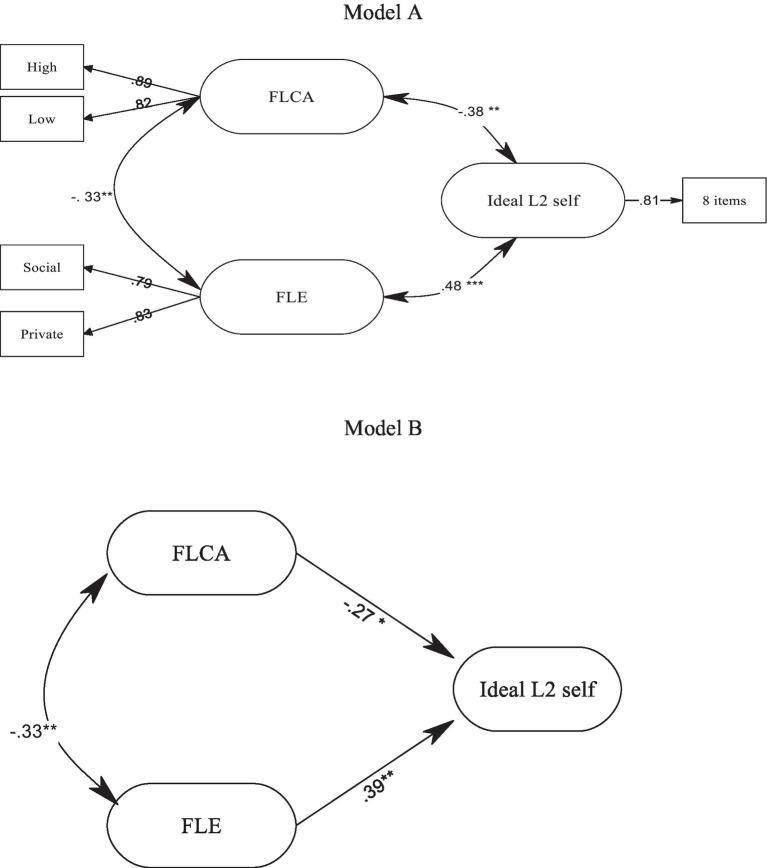
Foreign language classroom anxiety (FLCA) and foreign language enjoyment (FLE) as predictors of ideal L2 self. ^*^p<0.05, ^**^p<0.01, and ^***^p<0.001.

After that, SEM was utilized to test the structural model in which the two variables of FLCA and FLE acted as the predictors of ideal L2 self. To this end, two structural models were proposed, as depicted in Figure 1. The structures of the inter-connections for the two models (A and B) were the same. However, both models were tested to shed more light on the associations. Additionally, the unique contribution of each predictor (i.e., FLCA and FLE) on the criterion variable (i.e., ideal L2 self) was examined using fit indices and common variance measures. The models evaluated against fit indices showed a good fit for the structural models (see Table 3). As indicated in model A, FLE and FLCA had 11.2% of variance in common (*R*^2^=0.335). FLE and ideal L2 self showed 23.7% common variance (*R*^2^=0.487). Similarly, FLCA and ideal L2 self had 14.7% of shared variance (*R*^2^=0.384). Based on these results, it can be concluded that FLE was a stronger predictor of ideal L2 self than FLCA.

Furthermore, to determine the unique contribution of FLE and FLCA beyond and above each other, *R*^2^ increments were checked by comparing the percentage of variation in ideal L2 self, which is depicted in both models. Model B demonstrated that FLE and FLCA together explained 31% of the total variance in ideal L2 self. Thus, it can be stated that FLCA by itself contributed to the additional amount of 8% of the variance in ideal L2 self, beyond the single FLE (Δ*χ*^2^=0.31–0.23=0.08). Moreover, the unique contribution of FLE in accounting for ideal L2 self above the FLCA as the single explanatory variable was 20% (Δ*χ*^2^=0.31–0.11=0.20). As proved by these results, the unique impact of FLE was higher than FLCA (Table 4).

**Table 4:** Goodness of fit indices.

	*χ* ^2^	*χ*^2^/df	GFI	TLI	CFI	RMSEA	Δ*χ*^2^
Models A and B	5.36	1.72	0.98	0.97	0.98	0.042	
Model A1 (*β* FLCA=0)	10.62	2.53	0.97	0.98	0.97	0.035	5.26^*^
Model A2 (*β* FLE=0)	11.37	2.76	0.96	0.97	0.96	0.028	6.01^*^

* *p*<0.05.

Finally, the unique impacts of FLCA and FLE on ideal L2 self was tested by constraining each related beta weights to zero. The significant Δ*χ*^2^ was considered as the criterion for evaluating the models. In other words, in case zero beta weights leads to significant Δ*χ*^2^(i.e., changes in *χ*^2^); the unique impact of every predictor in the model is regarded to be substantial. Table 3 shows both the fit indices and Δ*χ*^2^ for the specified models. Constraining beta weights to zero for FLCA in model A1 (*β* FLCA=0) and anxiety in model A2 (*β* FLE=0) yielded significant Δ*χ*^2^ (model A1 (*β* FLCA=0): Δ*χ*^2^ (1, *N*=195)=5.26, *p*<0.01; model A2 (*β* FLE=0): Δ*χ*^2^ (1, *N*=195)=6.01, *p*<0.01). These results also verified the significant predictive power of FLCA and FLE in influencing ideal L2 self.

## Discussion

With the aim of shedding more light on the role of negative and positive emotions in L2 learning, the present study sought to explore the joint influence of negative and positive emotions on the ideal L2 self. More particularly, this research explored whether and how FLCA and FLE could predict ideal L2 self, a central element of L2 motivational self system (Dörnyei, 2009). The findings obtained from the proposed structural model indicated two major findings. First, it was revealed that FLCA predicted ideal L2 self negatively. This is partially in agreement with those of other empirical research (e.g., Papi, 2010; Yang, 2012; Ueki and Takeuchi, 2013), which reported a strong link between ideal L2 self and anxiety. The findings from a study of Yang (2012), for instance, showed that ideal L2 self acted as a significant correlate of FL anxiety. It was suggested that since Taiwanese students were highly motivated to achieve high proficiency level in English, they experienced higher anxiety level to reach their goal in the future. In other words, learners’ desires to become like native speakers caused them to feel a significant level of anxiety. Moreover, this is partially in line with the study conducted by Papi (2010), in which the impact of Iranian EFL students’ ideal L2 self on anxiety revealed that ideal L2 self had a strong role in decreasing their anxiety level.

In light of justifying such findings, it can be claimed that the high level of anxiety that learners experience prevents them from articulating answers to questions, following the lesson, and socializing with others. Therefore, given their inability in communication and learning, they visualize a negative and unclear self-image of their L2 learning in future. This argument can be at variance with Ueki and Takeuchi (2013), who suggested that students without a clear image of their ideal L2 self show greater tendency to deal with more anxiety in L2 learning compared to those who have clearer images. Moreover, it can be argued that students susceptible to high degrees of anxiety get demotivated when they are negatively evaluated because of their mistakes and poor performance. Therefore, the low levels of motivation dampen their desire and aspiration concerning language learning. This may suggest that FLCA has a detrimental influence on EFL students’ ideal self-images of L2 learning and its use in the future.

The second and foremost finding of this study revealed that FLE plays a positive stronger role in predicting ideal L2 self. This result resonates with the findings of a number of studies highlighting the correlation between FLE and ideal L2 self (Teimouri, 2017; Papi and Khajavy, 2021). The stronger effect of FLE on ideal L2 self can be justified in the light of Dewaele and MacIntyre (2014) findings, which indicated that students showed more enjoyment than anxiety in the classroom. Likewise, the results from the study of Khajavy et al. (2018) showed that Iranian EFL students experienced higher levels of enjoyment than anxiety inside the class. Given the results of these two studies, an interpretation seems valid: the more students enjoy the process of their learning, the less anxiety they experience. As a result, the high levels of students’ enjoyment and interest boost their motivation (Papi et al., 2019) which results in positive perceptions toward L2 self-images (e.g., attempting to speak the target language fluently) in the future. It is acknowledged that there exists a strong relationship between language learning enjoyment and motivation (see Zhang et al., 2020). This partially aligns with the investigation of Tahmouresi and Papi (2021), which indicated that ideal L2 writing self significantly affected L2 writing enjoyment, achievement, and motivation, thereby reducing their L2 writing anxiety. Further research carried out by Papi and Khajavy (2021) supported the significance of ideal L2 self in predicting enjoyment and anxiety. In a same vein, Teimouri (2017) found that the ideal L2 self and enjoyment were significantly associated.

The introduction of positive psychology in applied linguistics has caused a shift from exclusive concentration on negative emotions, particularly FLCA to positive emotions, such as FLE (Dewaele et al., 2019). Shifting from the only focus on negative emotions, researchers have shown a more integrated view toward negative as well as positive emotions that students experience in L2 learning process (Li et al., 2020). As Dewaele and MacIntyre (2016) have highlighted the influential role of both positive and negative emotions in language learning, suggesting that they are language leaners’ right and left feet. Research into positive emotions and negative emotions such as FLE and FLCA in FL classroom has flourished during the past decades. L2 researchers have tried to advance the existing knowledge on how FLCA as negative and FLE as positive emotions play significant roles in affecting learning from a dynamic perspective. However, considering the fact that no study has investigated the role of both FLCA and FLE in predicting language learners’ ideal L2 self-images, the present study attempted to fill this gap. The obtained findings of this study can enrich literature on how negative and positive emotions affect ideal L2 self, a core aspect of theory of motivation of Dörnyei (2005, 2009).

## Conclusion and Implications

Taken together, the findings of this study manifested the significant roles that FLCA and FLE play in learners’ ideal L2 self in an Iranian EFL setting. It was revealed that FLCA negatively predicted the ideal L2 self of EFL students, whereas FLE positively predicted ideal L2 self. This is to argue that students with greater enjoyment levels are likely to have positive image of their L2 self, while anxious students seem to have negative perceptions toward their ideal L2 self in the future. The results drawn from the present research might add novel insights to the extant related knowledge. These findings validate previous studies in the field of L2 learning which have highlighted the importance of students’ emotions and affective domain on their language learning motivation.

The findings of this study point to a number of potential pedagogical implications for teachers. EFL teachers can build a stress-free and comfortable environment by implementing various procedures, which promote students’ emotional security. In case teachers are able to lessen anxiety and increase enjoyment in learning atmosphere, they can enhance students’ ideal L2 self and their language learning motivation. Research has testified that language teachers have significant roles in decreasing FLCA and strengthening FLE among their students in the classrooms (Dewaele and Dewaele, 2020). From Dewaele and MacIntyre (2014) perspectives, in addition to friendly peers, and positive classroom environment, enjoyment is boosted by supportive teachers. There might be various practical strategies for teachers to create non-threatening classroom environments. For instance, allowing students to work in groups or pairs rather than lecturing alone in front of their peers can reduce their anxiety level. In addition, playing a short part of a musical instrument or song during break times or pumping students up with a 5-min challenge or physical activity, such as a simple whole-body stretches can be considered as relaxation exercises. These exercises can also give students feeling of enjoyment. For group discussions, teachers can also choose interesting and familiar topics, which students have background knowledge to talk about. Research has shown that students get nervous when they encounter unfamiliar topics (Cao and Philp, 2006). In another study, Pawlak and Mystkowska-Wiertelak (2015) maintained that students are unwilling to communicate when the topic is boring and tiring. Additionally, it is recommended that teachers use technology as a tool to teach a part of their lesson in order to make the learning more enjoyable and motivating. As an example, the teacher can divide the whole class into some groups consisting of 3–6 members and engage them in online tasks which they can work on collaboratively. The key is combining learning models and teaching practices with safe social media and technology learning tool use to promote students’ understanding.

Despite the fact that the present study yielded important results with regard to the effects of both negative and positive emotional factors (FLE and FLCA) on the students’ ideal L2 self, it also has some limitations. First, the relevant data were gathered from a relatively limited number of EFL learners in Iran. Thus, the obtained results may lack adequate generalizability to other contexts. In other words, every learning setting has its own particular culture and educational system that requires to be directly investigated before drawing any conclusions. Conducting a study with a nation-wide sample can add to the transferability and validity of the findings. In addition, the present researchers employed only quantitative research method. Therefore, using qualitative approaches in conjunction with questionnaires would further our understanding and give us a vivid image of the role of individual variables on motivation. Exploring how negative and positive emotional factors interact with cognitive variables *via* the lens of ideal L2 selves can be an intriguing topic for future researchers interested in this area.

## Data Availability Statement

The raw data supporting the conclusions of this article will be made available by the authors, without undue reservation.

## Ethics Statement

The studies involving human participants were reviewed and approved by University of Kurdistan. The patients/participants provided their written informed consent to participate in this study.

## Author Contributions

All authors listed have made a substantial, direct and intellectual contribution to the work, and approved it for publication.

## Conflict of Interest

The authors declare that the research was conducted in the absence of any commercial or financial relationships that could be construed as a potential conflict of interest.

## Publisher’s Note

All claims expressed in this article are solely those of the authors and do not necessarily represent those of their affiliated organizations, or those of the publisher, the editors and the reviewers. Any product that may be evaluated in this article, or claim that may be made by its manufacturer, is not guaranteed or endorsed by the publisher.

## Footnotes

^1^ https://docs.google.com

## References

[ref1] Alamer A. Almulhim F. (2021). The interrelation between language anxiety and self-determined motivation; a mixed methods approach. Front. Educ. 6:618655. doi: 10.3389/feduc.2021.618655

[ref2] Arnold J. (ed.) (1999). Affect in Language Learning. Cambridge: Cambridge University Press.

[ref3] Ary D. Jacobs L. C. Irvine C. K. S. Walker D. (2018). Introduction to Research in Education. Belmont, CA: Cengage Learning.

[ref4] Aslan E. Thompson A. S. (2021). The interplay between learner beliefs and foreign language anxiety: insights from the Turkish EFL context. Lang. Learn. J. 49, 189–202. doi: 10.1080/09571736.2018.1540649

[ref5] Blumberg P. Pontiggia L. (2011). Benchmarking the degree of implementation of learner-centered approaches. Innov. High. Educ. 36, 189–202. doi: 10.1007/s10755-010-9168-2

[ref6] Botes E. Dewaele J. M. Greiff S. (2020). The foreign language classroom anxiety scale and academic achievement: an overview of the prevailing literature and a meta-analysis. J. Psychol. Lang. Learn. 2, 26–56. doi: 10.52598/jpll/2/1/3

[ref7] Cao Y. (2014). A sociocognitive perspective on second language classroom willingness to communicate. TESOL Q. 48, 789–814. doi: 10.1002/tesq.155

[ref8] Cao Y. Philp J. (2006). Interactional context and willingness to communicate: a comparison of behavior in whole class, group and dyadic interaction. System 34, 480–493. doi: 10.1016/j.system.2006.05.002

[ref9] Chen T. Y. Chang G. B. (2004). The relationship between foreign language anxiety and learning difficulties. Foreign Lang. Ann. 37, 279–289. doi: 10.1111/j.1944-9720.2004.tb02200.x

[ref10] Derakhshan A. Kruk M. Mehdizadeh M. Pawlak M. (2021). Boredom in online classes in the Iranian EFL context: sources and solutions. System 101:102556. doi: 10.1016/j.system.2021.102556

[ref11] Dewaele J. M. (2017). “Psychological dimensions and foreign language anxiety,” in The Routledge Handbook of Instructed Second Language Acquisition. eds. S. Loewen and M. Sato (London : Routledge), 433–450.

[ref12] Dewaele J. M. (2019). The effect of classroom emotions, attitudes toward English, and teacher behavior on willingness to communicate among English foreign language learners. J. Lang. Soc. Psychol. 38, 523–535. doi: 10.1177/0261927X19864996

[ref13] Dewaele J. M. Alfawzan M. (2018). Does the effect of enjoyment outweigh that of anxiety in foreign language performance? Stud. Second Lang. Learn. Teach. 8, 21–45. doi: 10.14746/ssllt.2018.8.1.2

[ref14] Dewaele J. M. Chen X. Padilla A. M. Lake J. (2019). The flowering of positive psychology in foreign language teaching and acquisition research. Front. Psychol. 10:2128. doi: 10.3389/fpsyg.2019.02128, PMID: 31607981 PMC6769100

[ref15] Dewaele J. M. Dewaele L. (2017). The dynamic interactions in foreign language classroom anxiety and foreign language enjoyment of pupils aged 12 to 18. A pseudo-longitudinal investigation. J. Eur. Second Lang. Assoc. 1, 12–22. doi: 10.22599/jesla.6

[ref16] Dewaele J. M. Dewaele L. (2018). Learner-internal and learner-external predictors of willingness to communicate in the FL classroom. J. Eur. Second Lang. Assoc. 2, 24–37. doi: 10.22599/jesla.37

[ref17] Dewaele J. M. Dewaele L. (2020). Are foreign language learners’ enjoyment and anxiety specific to the teacher? An investigation into the dynamics of learners’ classroom emotions. Stud. Second Lang. Learn. Teach. 10, 45–65. doi: 10.14746/ssllt.2020.10.1.3

[ref19] Dewaele J. M. MacIntyre P. D. (2014). The two faces of Janus? Anxiety and enjoyment in the foreign language classroom. Stud. Second Lang. Learn. Teach. 4, 237–274. doi: 10.14746/ssllt.2014.4.2.5

[ref20] Dewaele J. M. MacIntyre P. D. (2016). “Foreign language enjoyment and foreign language classroom anxiety. The right and left feet of FL learning?” in Positive Psychology in SLA. eds. MacIntyre P. D. Gregersen T. Mercer S. (Bristol, UK: Multilingual Matters), 215–236.

[ref21] Dewaele J. M. MacIntyre P. D. (2019). “The predictive power of multicultural personality traits, learner and teacher variables on foreign language enjoyment and anxiety,” in Evidence-Based Second Language Pedagogy: A Collection of Instructed Second Language Acquisition Studies. eds. M. Sato and S. Loewen (London: Routledge), 263–286.

[ref22] Dewaele J. M. MacIntyre P. D. Boudreau C. Dewaele L. (2016). Do girls have all the fun? Anxiety and enjoyment in the foreign language classroom. Theory Pract. Second Lang. Acquis. 2, 41–63.

[ref23] Dewaele J. M. Magdalena A. F. Saito K. (2019). The effect of perception of teacher characteristics on Spanish EFL learners’ anxiety and enjoyment. Mod. Lang. J. 103, 412–427. doi: 10.1111/modl.12555

[ref24] Dewaele J. M. Pavelescu L. M. (2021). The relationship between incommensurable emotions and willingness to communicate in English as a foreign language: a multiple case study. Innov. Lang. Learn. Teach. 15, 66–80. doi: 10.1080/17501229.2019.1675667

[ref25] Dewaele J. M. Witney J. Saito K. Dewaele L. (2018). Foreign language enjoyment and anxiety in the FL classroom: the effect of teacher and learner variables. Lang. Teach. Res. 22, 676–697. doi: 10.1177/1362168817692161

[ref601] Dörnyei Z. (2005). The psychology of the language learner: Individual differences in second language acquisitions. Lawrence Erlbaum.

[ref26] Dörnyei Z. (2009). “Chapter 2: The L2 motivational self system,” in Motivation, Language Identity and the L2 Self. eds. Z. Dörnyei and E. Ushioda (Bristol: Multilingual Matters), 9–42.

[ref27] Dörnyei Z. (2010). “Researching motivation: from integrativeness to the ideal L2 self,” in Introducing Applied Linguistics: Concepts and Skills. eds. S. Hunston and D. Oakey (London: Routledge), 74–83.

[ref28] Dörnyei Z. Chan L. (2013). Motivation and vision: an analysis of future L2 self images, sensory styles, and imagery capacity across two target languages. Lang. Learn. 63, 437–462. doi: 10.1111/lang.12005

[ref29] Elahi-Shirvan M. Khajavy G. H. Nazifi M. Taherian T. (2018). Longitudinal examination of adult students’ self-efficacy and anxiety in the course of general English and their prediction by ideal self-motivation: latent growth curve modeling. New Horizons in Adult Education and Human Resource Development 30, 23–41. doi: 10.1002/nha3.20230

[ref30] Elahi-Shirvan M. Taherian T. (2018). Longitudinal examination of university students’ foreign language enjoyment and foreign language classroom anxiety in the course of general English: latent growth curve modeling. Int. J. Biling. Educ. Biling. 24, 31–49. doi: 10.1080/13670050.2018.1441804

[ref31] Emaliana I. (2017). Teacher-centered or student-centered learning approach to promote learning? J. Sosial Hum. 10, 59–70. doi: 10.12962/j24433527.v10i2.2161

[ref32] Esra M. E. Ş. E. Sevilen Ç. (2021). Factors influencing EFL students’ motivation in online learning: a qualitative case study. J. Educ. Technol. and Online Learning 4, 11–22.

[ref33] Eysenck M. W. Derakshan N. Santos R. Calvo M. G. (2007). Anxiety and cognitive performance: attentional control theory. Emotion 7:336. doi: 10.1037/1528-3542.7.2.336 17516812

[ref34] Feng L. Papi M. (2020). Persistence in language learning: the role of grit and future self-guides. Learn. Individ. Differ. 81:101904. doi: 10.1016/j.lindif.2020.101904

[ref35] Gardner R. C. (1985). Social Psychology and Second Language Learning: The Role of Attitudes and Motivation. London: Edward Arnold.

[ref36] Gardner R. C. Lambert W. E. (1972). Attitudes and Motivation in Second Language Learning. Massachusetts: Newbury House Publisher.

[ref37] Gregersen T. MacIntyre P. (2014). Capitalizing on Individual Differences: From Premise to Practice, Bristol: Multilingual Matters.

[ref38] Gregersen T. MacIntyre P. D. Meza M. D. (2014). The motion of emotion: idiodynamic case studies of learners' foreign language anxiety. Mod. Lang. J. 98, 574–588. doi: 10.1111/modl.12084

[ref39] Hismanoglu M. (2000). Language learning strategies in foreign language learning and teaching. Internet TESL J. 6:12

[ref40] Horwitz E. (2001). Language anxiety and achievement. Annu. Rev. Appl. Linguist. 21, 112–126. doi: 10.1017/S0267190501000071

[ref41] Horwitz E. K. (2010). Foreign and second language anxiety. Lang. Teach. 43, 154–167. doi: 10.1017/S026144480999036X

[ref42] Horwitz E. K. Horwitz M. B. Cope J. (1986). Foreign language classroom anxiety. Mod. Lang. J. 70, 125–132. doi: 10.1111/j.1540-4781.1986.tb05256.x

[ref43] Hu L. T. Bentler P. M. (1999). Cutoff criteria for fit indexes in covariance structure analysis: conventional criteria versus new alternatives. Struct. Equ. Model. Multidiscip. J. 6, 1–55. doi: 10.1080/10705519909540118

[ref44] Hu X. Zhang X. McGeown S. (2021). Foreign language anxiety and achievement: a study of primary school students learning English in China. Lang. Teach. Res. doi: 10.1177/13621688211032332 [Epub ahead of print]

[ref45] Jiang Y. Dewaele J. M. (2019). How unique is the foreign language classroom enjoyment and anxiety of Chinese EFL learners? System 82, 13–25. doi: 10.1016/j.system.2019.02.017

[ref46] Jiang Y. Dewaele J. M. (2020). The predictive power of sociobiographical and language variables on foreign language anxiety of Chinese university students. System 89:102207. doi: 10.1016/j.system.2020.102207

[ref47] Jiang G. Li C. (2017). SLA research in the positive psychology perspective: review and prospects. Foreign Lang. World 5, 32–39.

[ref48] Jin Y. Zhang L. J. (2018). The dimensions of foreign language classroom enjoyment and their effect on foreign language achievement. Int. J. Biling. Educ. Biling. 24, 948–962. doi: 10.1080/13670050.2018.1526253

[ref49] Khajavy G. H. Ghonsooly B. (2017). Predictors of willingness to read in English: testing a model based on possible selves and self-confidence. J. Multiling. Multicult. Dev. 38, 871–885. doi: 10.1080/01434632.2017.1284853

[ref50] Khajavy G. H. MacIntyre P. D. Barabadi E. (2018). Role of the emotions and classroom environment in willingness to communicate: applying doubly latent multilevel analysis in second language acquisition research. Stud. Second. Lang. Acquis. 40, 605–624. doi: 10.1017/S0272263117000304

[ref51] Khamkhien A. (2010). Factors affecting language learning strategy reported usage by Thai and Vietnamese EFL learners. Electron. J. Foreign Lang. Teach. 7, 66–85. doi: 10.4304/jltr.1.6.757-764

[ref52] Khodadady E. Khajavy G. H. (2013). Exploring the role of anxiety and motivation in foreign language achievement: a structural equation modeling approach. Porta Linguarum: revista internacional de didáctica de las lenguas extranjeras, 269–286. doi: 10.30827/Digibug.20240

[ref53] Kim Y. K. Kim T. Y. (2012). Korean secondary school students’ L2 learning motivation: comparing L2 motivational self system with socio-educational model. Engl. Lang. Lit. Teach. 18, 115–132.

[ref54] Kim T. Y. Kim Y. K. (2014). A structural model for perceptual learning styles, the ideal L2 self, motivated behavior, and English proficiency. System 46, 14–27. doi: 10.1016/j.system.2014.07.007

[ref55] Kline R. B. (2011). Principles and Practice of Structural Equation Modeling. 3rd *Edn*. New York, NY: Guilford Press.

[ref56] Kong J. H. Han J. E. Kim S. Park H. Kim Y. S. Park H. (2018). L2 motivational self system, international posture and competitiveness of Korean CTL and LCTL college learners: a structural equation modeling approach. System 72, 178–189. doi: 10.1016/j.system.2017.11.005

[ref57] Lee J. H. (2018). The effects of short-term study abroad on L2 anxiety, international posture, and L2 willingness to communicate. J. Multiling. Multicult. Dev. 39, 703–714. doi: 10.1080/01434632.2018.1435666

[ref58] Li C. (2020). A positive psychology perspective on Chinese EFL students’ trait emotional intelligence, foreign language enjoyment and EFL learning achievement. J. Multiling. Multicult. Dev. 41, 246–263. doi: 10.1080/01434632.2019.1614187

[ref59] Li C. Dewaele J. M. Jiang G. (2020). The complex relationship between classroom emotions and EFL achievement in China. Appl. Linguist. Rev. 11, 485–510. doi: 10.1515/applirev-2018-0043

[ref60] Li C. Huang J. Li B. (2021). The predictive effects of classroom environment and trait emotional intelligence on foreign language enjoyment and anxiety. System 96:102393. doi: 10.1016/j.system.2020.102393

[ref61] Li C. Jiang G. Dewaele J. M. (2018). Understanding Chinese high school students’ foreign language enjoyment: validation of the Chinese version of the foreign language enjoyment scale. System 76, 183–196. doi: 10.1016/j.system.2018.06.004

[ref62] Liu M. Jackson J. (2008). An exploration of Chinese EFL learners’ unwillingness to communicate and foreign language anxiety. Mod. Lang. J. 92, 71–86. doi: 10.1111/j.1540-4781.2008.00687.x

[ref64] MacIntyre P. Gregersen T. (2012). “Affect: the role of language anxiety and other emotions in language learning,” in Psychology for Language Learning (London: Palgrave Macmillan), 103–118.

[ref65] MacIntyre P. D. Mercer S. (2014). Introducing positive psychology to SLA. Stud. Second Lang. Learn. Teach. 4, 153–172. doi: 10.14746/ssllt.2014.4.2.2

[ref66] MacIntyre P. D. Noels K. A. Clément R. (1997). Biases in self-ratings of second language proficiency: the role of language anxiety. Lang. Learn. 47, 265–287. doi: 10.1111/0023-8333.81997008

[ref67] Magid M. Chan L. (2012). Motivating English learners by helping them visualise their ideal L2 self: lessons from two motivational programmes. Innov. Lang. Learn. Teach. 6, 113–125. doi: 10.1080/17501229.2011.614693

[ref68] Nomnian S. Arphattananon T. (2018). A qualitative study on factors influencing achievement of English language teaching and learning in Thai government secondary schools. Asian EFL J. 20, 207–233. doi: 10.22492/ije.6.2.04

[ref69] Nourzadeh S. Fathi J. Davari H. (2020). An examination of Iranian learners’ motivation for and experience in learning Korean as an additional language. Int. J. Multiling. 1–15. doi: 10.1080/14790718.2020.1850736 [Epub ahead of print]

[ref70] Papi M. (2010). The L2 motivational self system, L2 anxiety, and motivated behavior: a structural equation modeling approach. System 38, 467–479. doi: 10.1016/j.system.2010.06.011

[ref71] Papi M. Abdollahzadeh E. (2012). Teacher motivational practice, student motivation, and possible L2 selves: an examination in the Iranian EFL context. Lang. Learn. 62, 1–24. doi: 10.1111/j.1467-9922.2011.00632.x

[ref72] Papi M. Bondarenko A. V. Mansouri S. Feng L. Jiang C. (2019). Rethinking L2 motivation research: the 2× 2 model of L2 self-guides. Stud. Second. Lang. Acquis. 41, 337–361. doi: 10.1017/S0272263118000153

[ref73] Papi M. Khajavy G. H. (2021). Motivational mechanisms underlying second language achievement: a regulatory focus perspective. Lang. Learn. 71, 537–572. doi: 10.1111/lang.12443

[ref74] Pawlak M. Mystkowska-Wiertelak A. (2015). Investigating the dynamic nature of L2 willingness to communicate. System 50, 1–9. doi: 10.1016/j.system.2015.02.001

[ref75] Peng J. E. (2015). L2 motivational self system, attitudes, and affect as predictors of L2 WTC: an imagined community perspective. Asia Pac. Educ. Res. 24, 433–443. doi: 10.1007/s40299-014-0195-0

[ref76] Petrides J. R. (2006). Attitudes and motivation and their impact on the performance of young English as a foreign language learners. J. Lang. Learn. 5, 1–20.

[ref77] Pichette F. (2009). Second language anxiety and distance language learning. Foreign Lang. Ann. 42, 77–93. doi: 10.1111/j.1944-9720.2009.01009.x

[ref78] Pishghadam R. Derakhshan A. Zhaleh K. Al-Obaydi L. H. (2021). Students’ willingness to attend EFL classes with respect to teachers’ credibility, stroke, and success: a cross-cultural study of Iranian and Iraqi students’ perceptions. Curr. Psychol. 40, 1–15. doi: 10.1007/s12144-021-01738-z

[ref79] Russell V. (2020). Language anxiety and the online learner. Foreign Lang. Ann. 53, 338–352. doi: 10.1111/flan.12461

[ref80] Ryan S. (2009). “Chapter 6: self and identity in L2 motivation in Japan: the ideal L2 self and Japanese learners of English,” in Motivation, Language Identity and the L2 Self. eds. Z. Dörnyei and E. Ushioda (Bristol, UK: Multilingual Matters), 120–143.

[ref81] Sadoughi M. Hejazi S. Y. (2021). Teacher support and academic engagement among EFL learners: the role of positive academic emotions. Stud. Educ. Eval. 70:101060. doi: 10.1016/j.stueduc.2021.101060

[ref82] Seligman M. E. Csikszentmihalyi M. (2000). Positive psychology: an introduction. Am. Psychol. 55, 5–14. doi: 10.1037/0003-066X.55.1.5 11392865

[ref83] Tabachnick B. G. Fidell L. S. (2007). Using Multivariate Statistics. 5th *Edn*. Boston, MA: Pearson Education.

[ref84] Taguchi T. Magid M. Papi M. (2009). “Chapter 4: The L2 motivational self system among Japanese, Chinese and Iranian learners of English: a comparative study,” in Motivation, Language Identity and the L2 Self. eds. Z. Dörnyei and E. Ushioda (Bristol, UK: Multilingual Matters), 66–97.

[ref85] Tahmouresi S. Papi M. (2021). Future selves, enjoyment and anxiety as predictors of L2 writing achievement. J. Second. Lang. Writ. 53:100837. doi: 10.1016/j.jslw.2021.100837

[ref86] Teimouri Y. (2017). L2 selves, emotions, and motivated behaviors. Stud. Second. Lang. Acquis. 39, 681–709. doi: 10.1017/S0272263116000243

[ref87] Teimouri Y. Goetze J. Plonsky L. (2019). Second language anxiety and achievement: a meta-analysis. Stud. Second. Lang. Acquis. 41, 363–387. doi: 10.1017/S0272263118000311

[ref88] Teimouri Y. Plonsky L. Tabandeh F. (2020). L2 grit: passion and perseverance for second-language learning. Lang. Teach. Res. 1–26. doi: 10.1177/1362168820921895

[ref89] Ueki M. Takeuchi O. (2013). Exploring the concept of the ideal L2 self in an Asian EFL context: the case of Japanese university students. J. Asia TEFL 10, 25–45.

[ref90] Wang Y. Derakhshan A. Zhang L. J. (2021). Researching and practicing positive psychology in second/foreign language learning and teaching: the past, current status and future directions. Front. Psychol. 12:731721. doi: 10.3389/fpsyg.2021.731721, PMID: 34489835 PMC8417049

[ref91] Yang H. C. (2012). Language anxiety, acculturation, and L2 self: A relational analysis in the Taiwanese cultural context. Electron. J. Foreign Lang. Teach. 9, 183–193.

[ref92] Yashima T. (2009). “Chapter 7: international posture and the ideal L2 self in the Japanese EFL context,” in Motivation, Language Identity and the L2 Self (Multilingual Matters), 144–163.

[ref93] Yousefifard S. Fathi J. (2021). Exploring the impact of blogging in English classrooms: focus on the ideal writing self of EFL learners. Int. J. Instr. 14, 913–932. doi: 10.29333/iji.2021.14452a

[ref94] Zarrinabadi N. Lou N. M. Shirzad M. (2021). Autonomy support predicts language mindsets: implications for developing communicative competence and willingness to communicate in EFL classrooms. Learn. Individ. Differ. 86:101981. doi: 10.1016/j.lindif.2021.101981

[ref95] Zeng G. Hou H. Peng K. (2016). Effect of growth mindset on school engagement and psychological well-being of Chinese primary and middle school students: the mediating role of resilience. Front. Psychol. 7:1873. doi: 10.3389/fpsyg.2016.01873, PMID: 28018251 PMC5147462

[ref96] Zhang H. Dai Y. Wang Y. (2020). Motivation and second foreign language proficiency: the mediating role of foreign language enjoyment. Sustainability 12:1302. doi: 10.3390/su12041302

[ref97] Zhou L. Xi Y. Lochtman K. (2020). The relationship between second language competence and willingness to communicate: the moderating effect of foreign language anxiety. J. Multiling. Multicult. Dev. 43, 1–15. doi: 10.1080/01434632.2020.1801697

